# Directed Differentiation of Mobilized Hematopoietic Stem and Progenitor Cells into Functional NK Cells with Enhanced Antitumor Activity

**DOI:** 10.3390/cells9040811

**Published:** 2020-03-27

**Authors:** Pranav Oberoi, Kathrina Kamenjarin, Jose Francisco Villena Ossa, Barbara Uherek, Halvard Bönig, Winfried S. Wels

**Affiliations:** 1 Georg-Speyer-Haus, Institute for Tumor Biology and Experimental Therapy, 60596 Frankfurt am Main, Germany; kathrina@kamenjarin.de (K.K.); francisco.villenaossa@kgu.de (J.F.V.O.); uherek@gsh.uni-frankfurt.de (B.U.); wels@gsh.uni-frankfurt.de (W.S.W.); 2 Frankfurt Cancer Institute, Goethe University, 60590 Frankfurt am Main, Germany; 3 German Cancer Consortium (DKTK), Partner Site Frankfurt/Mainz, 60590 Frankfurt am Main, Germany; 4 German Cancer Research Center (DKFZ), 69120 Heidelberg, Germany; 5 Goethe University, Institute for Transfusion Medicine and Immunohematology, and German Red Cross Blood Donation Service Baden-Württemberg - Hessen, 60528 Frankfurt am Main, Germany; h.boenig@blutspende.de

**Keywords:** natural killer cells, hematopoietic stem and progenitor cells, feeder cells, natural cytotoxicity, adoptive cancer immunotherapy

## Abstract

Obtaining sufficient numbers of functional natural killer (NK) cells is crucial for the success of NK-cell-based adoptive immunotherapies. While expansion from peripheral blood (PB) is the current method of choice, ex vivo generation of NK cells from hematopoietic stem and progenitor cells (HSCs) may constitute an attractive alternative. Thereby, HSCs mobilized into peripheral blood (PB-CD34^+^) represent a valuable starting material, but the rather poor and donor-dependent differentiation of isolated PB-CD34^+^ cells into NK cells observed in earlier studies still represents a major hurdle. Here, we report a refined approach based on ex vivo culture of PB-CD34^+^ cells with optimized cytokine cocktails that reliably generates functionally mature NK cells, as assessed by analyzing NK-cell-associated surface markers and cytotoxicity. To further enhance NK cell expansion, we generated K562 feeder cells co-expressing 4-1BB ligand and membrane-anchored IL-15 and IL-21. Co-culture of PB-derived NK cells and NK cells that were ex-vivo-differentiated from HSCs with these feeder cells dramatically improved NK cell expansion, and fully compensated for donor-to-donor variability observed during only cytokine-based propagation. Our findings suggest mobilized PB-CD34^+^ cells expanded and differentiated according to this two-step protocol as a promising source for the generation of allogeneic NK cells for adoptive cancer immunotherapy.

## 1. Introduction

Natural killer (NK) cells are innate lymphocytes that play a crucial role in defense against viral infections and cancer immunosurveillance. Unlike B and T cells, NK cells do not require prior sensitization, specific antigen recognition, and clonal expansion. Instead, their natural cytotoxicity can be triggered rapidly, and is regulated by a complex balance of signals from germline-encoded activating and inhibitory cell surface receptors [1,2]. Healthy cells are protected against NK-mediated lysis through surface expression of “self” major histocompatibility complex (MHC) class I molecules, which engage with inhibitory receptors on NK cells and block their effector functions. In contrast, transformed cells frequently downregulate MHC class I molecules, and in addition upregulate stress-induced ligands, such as MHC class I polypeptide-related sequence A/B (MICA/B), enabling their recognition and subsequent lysis by NK cells [3,4]. Due to this intrinsic ability to efficiently detect and eliminate malignant cells, NK cells are promising effectors for adoptive cancer immunotherapy [5,6].

A prerequisite for successful NK-cell-based therapy is to obtain sufficient effector cell numbers with high purity and antitumor activity. Different approaches for large-scale ex vivo NK cell expansion have been reported [7,8,9]. Usually, NK cells are isolated from peripheral blood mononuclear cells (PBMCs) by leukapheresis, and more recently also cord blood (CB) mononuclear cells, and expanded and activated ex vivo with cytokines such as interleukin (IL)-2 and IL-15 before infusion into patients [10,11]. With respect to purity, this process is rather effective, regularly yielding >90% of CD56^+^ NK cells. However, the cell numbers obtained are often not enough to achieve clinical benefits. Hence, to enhance NK cell proliferation and activity ex vivo, several studies have employed feeder cells such as autologous PBMCs, virus-transformed lymphoblastoid cell lines, or genetically-engineered K562 leukemia cells, which can stimulate NK cell expansion through humoral signals and cell-to-cell contact [12,13,14,15,16,17,18]. The combination of irradiated feeder cells and low-dose cytokines has been shown to be more effective in inducing NK cell expansion, and this stimulatory effect can be sustained over prolonged periods of continuous culture.

Additionally, ex vivo differentiation of stem cells from different sources has been used to generate CD56^+^ NK cells, including hematopoietic stem and progenitor cells (HSCs) from bone marrow and cord blood, human embryonic stem cells (hESCs), and induced pluripotent stem cells (iPSCs) [19,20,21,22]. Among these, cord blood HSCs have emerged as a preferred source of CD34^+^ cells due to their primitive stem cell characteristics and high proliferative capacity. Several groups have reported efficient generation of CB-CD34^+^-derived NK cell products that display enhanced effector functions and cytolytic activity against tumor cells in vitro and in vivo [20,22,23,24]. In a phase I clinical trial, infusions of CB-CD34^+^-derived NK cells in acute myeloid leukemia (AML) patients were well tolerated, encouraging further development of this approach [25]. Likewise, iPSC-derived NK cells with antitumor activity can be manufactured at a clinical scale [26,27,28].

Granulocyte colony-stimulating factor (G-CSF)-mobilized HSCs isolated from peripheral blood of healthy donors (PB-CD34^+^) represent another valuable starting material for ex vivo generation of NK cells. PB-CD34^+^ cells are easy to access, and can usually be collected at higher quantities than CB-CD34^+^ cells. However, in comparison to cord blood HSCs, ex vivo NK cell generation from PB-CD34^+^ cells is more challenging. Earlier studies have reported that differentiation of mobilized HSCs from peripheral blood into NK cells is often poor, with decreased viability, low activity, and high donor-to-donor variability [29,30,31,32]. For these reasons, here we developed a refined approach for ex vivo differentiation of PB-CD34^+^ cells into functional NK cells based on sequential cultivation in optimized cytokine cocktails. The resulting CD56^+^ NK cell products were characterized by investigating surface expression of NK-cell-associated activating and inhibitory receptors, and cytotoxicity against tumor cell lines. To address remaining donor-to-donor variability with respect to the extent of differentiation and cytolytic activity of the resulting CD56^+^ NK cells, we introduced a subsequent cultivation step by exposing the differentiating cells to K562 feeder cells, which we engineered to co-express membrane-anchored pro-NK-cell factors, including IL-15 and IL-21.

## 2. Materials and Methods

### 2.1. Cells and Culture Conditions

K562 erythroleukemia, Raji Burkitt’s lymphoma and DU145 prostate cancer cells (all ATCC, Manassas, VA) were maintained in RPMI 1640 medium (Gibco, Thermo Fischer Scientific, Bonn, Germany). Likewise, K562-mb15-41BBL feeder cells (designated here as K562/41BBL/mb15; kindly provided by Helmut Salih, University of Tübingen) were cultured and maintained in RPMI 1640 medium as previously described [12]. MDA-MB453 breast carcinoma, MDA-MB435 melanoma, A431 squamous cell carcinoma, LN-18 glioblastoma, and HEK 293T embryonic kidney cells (all ATCC) were cultured in DMEM (Gibco, Thermo Fischer Scientific). All media were supplemented with 10% heat-inactivated FBS, 2 mM L-glutamine, 100 U/mL penicillin, and 100 µg/mL streptomycin (Life Technologies, Darmstadt, Germany).

### 2.2. Selection of CD34^+^ Cells, Ex Vivo Expansion, and Differentiation into NK Cells

CD34^+^ hematopoietic stem and progenitor cells were isolated from leftover materials of G-CSF-mobilized apheresis products collected from healthy individuals who had donated cells for routine allogeneic stem cell transplantation purposes at the Institute for Transfusion Medicine and Immunohematology in Frankfurt. Mononuclear cells (MNCs) from the apheresis product were isolated by Ficoll–Hypaque (1.077 g/mL; Lonza, Cologne, Germany) density gradient centrifugation, and CD34^+^ cells were obtained by magnetic cell sorting using the CD34 MicroBead kit and autoMACS^®^ Pro Separator (both Miltenyi Biotec, Bergisch Gladbach, Germany), according to the manufacturer’s instructions. The purity of the enriched CD34^+^ cells was analyzed by flow cytometry and ranged between 92–96%. All human materials were collected and used after informed donor consent and by following the guidelines of the respective institutional ethics committee (Ethik-Kommission, Goethe University, Frankfurt, approval number 329/10).

For ex vivo expansion, typically 5 × 10^5^ purified CD34^+^ cells were cultured for 5 days in serum-free CellGro^®^ Stem Cell Growth Medium (SCGM) (Cell Genix, Freiburg, Germany) supplemented with 100 U/mL penicillin, 100 µg/mL streptomycin, recombinant human IL-6 (25 ng/mL), recombinant human thrombopoietin (TPO; 25 ng/mL), recombinant human stem cell growth factor (SCF; 30 ng/mL), and recombinant human fms-like tyrosine kinase 3 ligand (FLT3L; 50 ng/mL) (all from PeproTech, Hamburg, Germany). To facilitate generation and development of NK cells, starting at day 6 the ex-vivo-expanded CD34^+^ cells were transferred to a differentiation medium comprising CellGro^®^ SCGM supplemented with 100 U/mL penicillin, 100 µg/mL streptomycin, 5% (*v*/*v*) heat-inactivated human plasma, 10 μM hydrocortisone (Sigma-Aldrich, Taufkirchen, Germany), SCF (30 ng/mL), FLT3L (50 ng/mL), recombinant human IL-7 (50 ng/mL), recombinant human insulin-like growth factor 1 (IGF-1; 100 ng/mL), recombinant human IL-15 (50 ng/mL) (all PeproTech), and recombinant human IL-2 (500 IU/mL; Proleukin^®^, Novartis Pharma, Nürnberg, Germany). Cells were maintained at a density of 1 × 10^6^ and 2-3 × 10^6^ cells/mL during the expansion and differentiation phases, respectively, with half-medium change every 2–3 days.

### 2.3. Phenotyping of Ex-Vivo-Generated NK Cells

NK cells arising from CD34^+^ HSCs were characterized by detection of the lineage-specific marker CD56 throughout the differentiation process. Briefly, 2–2.5 × 10^5^ cells were harvested from the differentiation culture, washed once with Dulbecco’s phosphate-buffered saline (DPBS), and stained with BV421-conjugated anti-human CD56 antibody (BD Biosciences, Heidelberg, Germany) for 60 min at 4 °C. Then, cells were washed twice with DPBS and analyzed using a FACSCanto II flow cytometer and FACSDiva software (BD Biosciences). All staining procedures were performed in the presence of a human Fc receptor blocking agent (BD Biosciences). Unstained cells were included as controls. Additionally, cells from the differentiation culture were stained with allophycocyanin (APC)-conjugated CD3 antibody (BD Biosciences), fluorescein isothiocyanate (FITC)-conjugated CD19 antibody (Invitrogen, Thermo Fischer Scientific), APC-Vio770-conjugated CD11b antibody (Miltenyi Biotec), and phycoerythrin (PE)-conjugated CD14 antibody (BD Biosciences) to assess the development of T, B, and myeloid lineages, respectively.

Expression of NK-cell-associated surface markers was detected with Alexa Fluor 647- or APC-conjugated CD16 antibody, PE-conjugated CD337 (NKp30) antibody, Alexa Fluor 647-conjugated CD336 (NKp44) antibody (all BD Biosciences), PE-conjugated CD314 (NKG2D) antibody, PE-Vio770- or APC-conjugated CD335 (NKp46) antibody, PE-conjugated KIR2D antibody, APC-conjugated CD159a (NKG2A) antibody, and FITC-conjugated CD94 antibody (all Miltenyi Biotec) by flow cytometry, as described above.

### 2.4. Generation of K562/41BBL/mb15/mb21 Feeder Cells

A fusion construct comprising an immunoglobulin heavy chain signal peptide, codon-optimized full-length human IL-21, a Myc-tag, a modified CD8α hinge region (UniProt P01732; residues 117-178; cysteine 164 replaced by a serine) [33], and the transmembrane region of CD28 followed by a truncated CD28 intracellular domain (CD28.TM) (UniProt P10747; residues 151-185, followed by a proline residue and a stop codon) [34] was assembled in silico and de novo synthesized (GeneArt, Thermo Fischer Scientific). The IL-21-CD28.TM (21.TM) sequence was inserted into lentiviral transfer plasmid pHR’SIN-cPPT-SIRW (pSIRW) upstream of IRES and iRFP sequences of the vector, resulting in plasmid pS-21.TM-IRW, which allows co-expression of membrane-bound IL-21 (mb21) and near-infrared fluorescent protein (iRFP). Lentiviral transfer plasmid pSIRW is a modified version of the pSIEW construct [35], wherein the enhanced green fluorescent protein (EGFP) sequence of pSIEW was replaced with the sequence encoding iRFP (kindly provided by Sabrina Genßler, Georg-Speyer-Haus, Frankfurt).

VSV-G pseudotyped lentiviral vector particles were produced using HEK 293T cells as described [36]. For transduction, lentiviral particle-containing supernatant was added to EGFP-positive K562/41BBL/mb15 or parental K562 cells in the presence of 8 µg/mL polybrene, followed by centrifugation for 90 min at 32 °C and 1800× *g*. Then, the cells were cultured for 6 h at 37 °C before replacing the medium. After 72 h, cells were analyzed for iRFP expression by direct flow cytometry using a FACSCanto II flow cytometer and FACSDiva software. The iRFP and EGFP co-expressing K562/41BBL/mb15/mb21 and iRFP-expressing K562/mb21 feeder cells were obtained through two rounds of sorting using a FACSAria fluorescence-activated cell sorter (BD Biosciences).

### 2.5. Analysis of IL-21 Expression and Activity

Whole cell lysates of parental and gene-modified K562 feeder cells were prepared by sonication at 4 °C in buffer containing 50 mM Tris-HCl, pH 7.5, 150 mM NaCl, 5 mM EDTA, 0.5% sodium deoxycholate, 1% NP-40, 0.1% SDS (all Roth, Karlsruhe, Germany), 1 mM PMSF, 1 mM sodium orthovanadate (Sigma-Aldrich), and protease inhibitor cocktail (Roche Diagnostics, Mannheim, Germany). Subsequently, cleared cell extracts were subjected to SDS-PAGE and immunoblot analysis with a CD8α-specific antibody (Santa Cruz Biotechnology, Heidelberg, Germany), followed by horseradish peroxidase (HRP)-conjugated secondary antibody and chemiluminescent detection. As a loading control, blots were reprobed with γ-tubulin-specific antibody (Sigma-Aldrich). To examine IL-21 surface expression, K562/41BBL/mb15/mb21 cells (5 × 10^5^) were incubated with BV421-conjugated IL-21 antibody (BD Biosciences) for 60 min at 4 °C and analyzed with a FACSCanto II flow cytometer and FACSDiva software. Parental K562 and K562/41BBL/mb15 cells were included as controls. Surface expression of 4-1BB ligand (4-1BBL) and membrane-bound IL-15 on K562/41BBL/mb15 and K562/41BBL/mb15/mb21 cells was assessed with 4-1BBL antibody (clone 282220) and IL-15 antibody (clone 34559; both R&D Systems, Wiesbaden-Nordenstadt, Germany), followed by APC-conjugated goat anti-mouse secondary antibody (Jackson ImmunoResearch, West Grove, PA, USA) and flow cytometric analysis, as described above.

To investigate IL-21 activity, Raji cells (1.5 × 10^6^) were cultured overnight in serum-free medium and then stimulated with either K562/41BBL/mb15/mb21 cells (1.5 × 10^6^) or 10 ng/mL recombinant IL-21 (PeproTech) for 30 min at 37 °C. Subsequently, cell lysates were prepared and immunoblot analysis was performed with phospho-STAT3-specific antibody (Cell Signaling Technology, Frankfurt, Germany), followed by HRP-conjugated secondary antibody and chemiluminescent detection, as described above.

### 2.6. Cultivation of Peripheral Blood-Derived NK Cells

Peripheral blood mononuclear cells (PBMCs) were isolated from buffy coats of healthy donors by Ficoll–Hypaque density gradient centrifugation. For expansion of NK cells, PBMCs (1.5 × 10^6^) were then co-cultured with lethally irradiated (100 Gy) K562-derived feeder cells (1 × 10^6^) in 2 mL of RPMI 1640 medium supplemented with 10% heat-inactivated FBS, 2 mM l-glutamine, 100 U/mL penicillin, 100 µg/mL streptomycin, and 50 IU/mL recombinant human IL-2. Every 7 days, cells were counted and 1 × 10^6^ cells from the expanding cell pool were restimulated with 1 × 10^6^ irradiated feeder cells. Culture conditions were maintained for up to 4 weeks with half-medium change every 2–3 days. PBMCs cultured in medium without feeder cells served as controls. Expansion of CD56^+^ NK cells was analyzed every week by flow cytometry, as described above for ex-vivo-generated NK cells.

### 2.7. Co-Culture of CD34^+^ Cells with K562-Derived Feeder Cells

Four weeks after initiating the ex vivo CD34^+^ cell expansion and differentiation process, 2 × 10^6^ cells were harvested from the cell pool and co-cultured with 1 × 10^6^ lethally irradiated (100 Gy) K562-derived feeder cells in 2 mL of the regular differentiation medium. One week later (day 7 of feeder cell stimulation), cells were counted and analyzed by flow cytometry. If the proportion of feeder cells in the culture mix was <5% (determined as iRFP/EGFP double-positive for K562/41BBL/mb15/mb21), the expanding cell pools were restimulated with 1 × 10^6^ irradiated feeder cells. If feeder cell proportion was >5%, culture conditions were maintained for another week with half-medium change every 2–3 days, followed by restimulation with 1 × 10^6^ irradiated feeder cells on day 14 of the co-culture, with no further restimulation thereafter. For comparative analysis, CD34^+^ cells cultured in regular cytokine-containing medium without feeder cells were always included as a control. Development of CD56^+^ NK cells in the presence or absence of feeder cells was analyzed every week by flow cytometry as described before.

### 2.8. Cytotoxicity Assay

Cytotoxic activity of ex-vivo-generated and PBMC-expanded NK cells against target cells was analyzed in flow-cytometry-based assays as described [36]. Briefly, target cells were labeled with calcein violet AM (Molecular Probes, Invitrogen), washed, and then co-cultured with effector cells at various effector to target (E/T) ratios for 2.5 h at 37 °C. After co-culture, cells were centrifuged, and 200 µL of a 1 μg/mL propidium iodide (PI) solution were added to each sample shortly before flow cytometry analysis with a FACSCanto II flow cytometer. Specific cytotoxicity was calculated using FACSDiva software. Dead target cells were determined as calcein violet AM and PI double-positive. Spontaneous target cell lysis was analyzed in samples containing only labeled target cells and subtracted to obtain specific cell killing. For all cytotoxicity assays, the E/T ratio was calculated based on the proportion of CD56^+^ NK cells in the co-culture.

### 2.9. Statistical Analysis

Differences between values were evaluated using the two-tailed unpaired Student’s *t* test. The *p* values < 0.05 were considered significant. Statistical calculations were done using Prism 7 software (GraphPad Software, La Jolla, CA).

## 3. Results

### 3.1. Ex Vivo Expansion of Mobilized CD34^+^ Hematopoietic Progenitors and Differentiation into NK Cells

**Figure 1:**
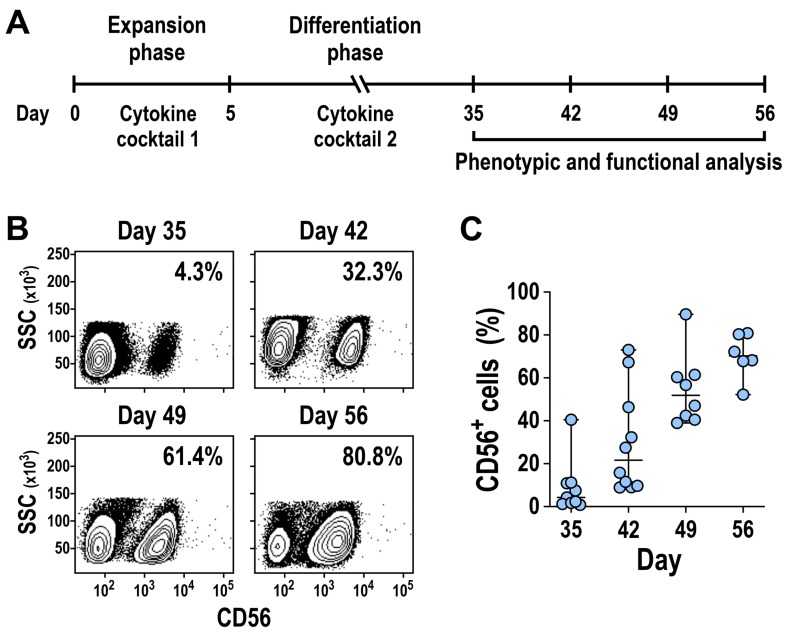
Ex vivo generation of natural killer (NK) cells from peripheral blood-derived CD34^+^ hematopoietic progenitors. (**A**) Schematic representation of the procedure used for ex vivo generation of NK cells from mobilized PB-CD34^+^ hematopoietic stem and progenitor cells (HSCs). The culture protocol comprises an initial expansion phase of up to 5 days in serum-free medium containing stem cell growth factor (SCF), thrombopoietin (TPO), interleukin (IL)-6, and fms-like tyrosine kinase 3 ligand (FLT3L) (cytokine cocktail 1), followed by a differentiation phase of about 4 weeks in serum-rich medium supplemented with SCF, IL-7, FLT3L, IGF-1, IL-15, IL-2, hydrocortisone, and human plasma (cytokine cocktail 2). (**B**,**C**) Development and expansion of CD56^+^ NK cells over time was analyzed by flow cytometry. A representative donor is shown in (**B**). Median CD56^+^ cell expansion (with range) using CD34^+^ cells from different donors is shown in (**C**).

To facilitate NK cell generation from HSCs ex vivo, we established a protocol based on sequential exposure of the cells to different cytokine cocktails (Figure 1A). Mobilized CD34^+^ cells isolated from the peripheral blood of healthy donors were first cultured for up to 5 days in an enriched serum-free medium containing IL-6, SCF, TPO, and FLT3L to allow acclimatization and expansion of the progenitor cells (expansion phase). Subsequently, the expanded cells were transferred to a serum-rich medium supplemented with a variety of pro-NK-cell cytokines, including SCF, FLT3L, IL-7, IL-2, and IL-15, to allow differentiation into NK cells (differentiation phase). In addition, IGF-1 was included, which has been shown to play a crucial role in regulating growth and function of NK cells [37]. To limit myeloid cell development during the differentiation process, hydrocortisone (HDC) was added to the culture mix [38,39].

Following this ex vivo differentiation scheme, we observed the appearance of a distinct population of CD56^+^ NK cells starting at day 35 of the culture, with the proportion of these cells in the differentiating cell pool rapidly increasing over time (Figure 1B). Differentiation experiments performed multiple times with mobilized PB-CD34^+^ cells from independent healthy donors yielded similar results (Figure 1C). Thereby, the median proportion of CD56^+^ cells among the cell populations derived from mobilized HSCs was 4.3% (range 0.8–40.5%; *n* = 9) at day 35, 21.7% (range 8.9–73%; *n* = 10) at day 42, 51.9% (range 39–89.6%; *n* = 8) at day 49, and 70.2% (range 52.2–80.8%; *n* = 6) at day 56. Importantly, we never detected B cells (CD19^+^) or T cells (CD3^+^) in the cultures (data not shown), demonstrating that this approach supports selective generation of NK cells. Following the same strategy, we could also generate CD56^+^ cells from bone-marrow-derived CD34^+^ cells (BM-CD34^+^) (Supplementary Figure S1A).

**Figure 2:**
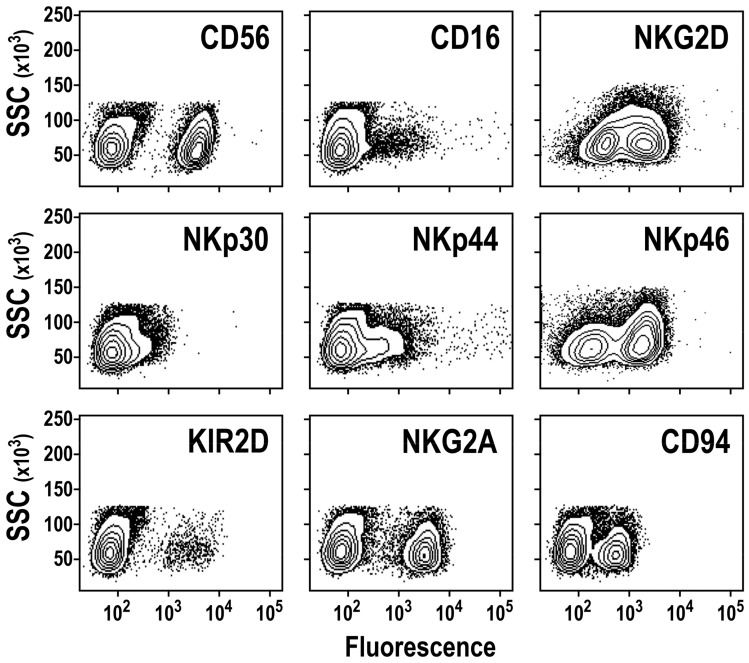
Phenotypic characterization of ex-vivo-generated CD56^+^ NK cells. Surface expression of NK-cell-associated activating receptors CD16, NKG2D and natural cytotoxicity receptors (NCRs; NKp30, NKp44, NKp46), inhibitory receptors NKG2A and CD94, and pan-KIR2D receptors (both activating and inhibitory) by CD56^+^ cells generated ex vivo from CD34^+^ cells was analyzed by flow cytometry after six to eight weeks of culture. Each plot is representative of at least five independent experiments.

Next, we performed phenotypic analysis of the ex-vivo-generated NK cells. After six to eight weeks in culture, the differentiated cells displayed high expression of the activating receptor NKG2D, while CD16 was either absent or expressed at relatively low levels, varying considerably between donors (Figure 2). The ex-vivo-generated NK cells stably expressed the natural cytotoxicity receptors (NCRs) NKp46 and NKp44, whereas NKp30 expression varied between donors from low to high (Figure 2; see also Figure 7A). Furthermore, we observed homogeneous and high expression of NKG2A and CD94, whereas only a small proportion of cells were positive for KIR2D (isoforms KIR2DL and KIR2DS). Similar results were observed for BM-CD34^+^-derived NK cells (Supplementary Figure S1B).

### 3.2. Donor-to-Donor Variation as a Limiting Factor for Ex Vivo NK Cell Generation

**Figure 3:**
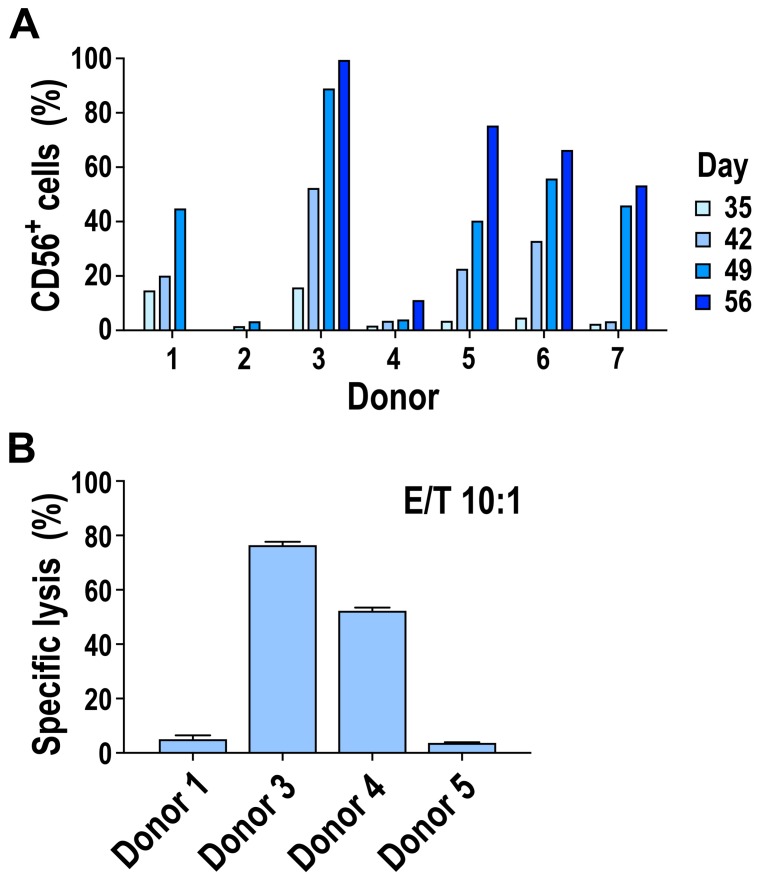
Donor-to-donor variation in the generation of CD56^+^ NK cells from mobilized PB-CD34^+^ cells. Mobilized HSCs from seven healthy donors were cultured ex vivo following the scheme depicted in Figure 1A. (**A**) Development of CD56^+^ NK cells was analyzed at the indicated time points of the culture period by flow cytometry. For donors 1 and 2, no data were available for day 56, or for days 35 and 56, respectively. (**B**) Cytotoxicity of CD56^+^ NK cells generated from PB-CD34^+^ cells was determined in flow-cytometry-based cell killing assays at an effector to target (E/T) ratio of 10:1. Mean values ± SEM of three technical replicates for each donor are shown. Identical donor numbers in (A) and (B) indicate the same donors.

Although we could reliably generate mature CD56^+^ NK cells from mobilized CD34^+^ cells, the extent of differentiation and functionality of the resulting NK cells varied considerably between donor CD34^+^ cells used as the starting material. Despite being cultured under the same conditions, the generation of NK cells appeared to be delayed for some donors, where a substantial CD56^+^ population was only detected starting at day 42 or 49 instead of day 35 of the culture period (Figure 3A). Furthermore, CD34^+^ progenitors from certain donors showed poor to no differentiation into CD56^+^ NK cells (Figure 3A, donors 2 and 4). Instead, in these cultures the majority of cells in the differentiating cell pool were CD11b^+^, and while they were negative for CD33, they expressed the myeloid markers CD13, CD14, and CD15, indicating preferential differentiation into the myeloid lineage (data not shown). This differential CD56^+^ cell expansion sometimes resulted in low yields and inhomogeneous cell populations, with the purity of the final NK cell product among the different donors tested covering a relatively wide range of 11.3–99.6% at day 56 of the culture period (see Figure 1C, Figure 3A).

To determine the functionality of the ex-vivo-generated NK cells, we performed flow-cytometry-based cytotoxicity assays using K562 leukemia cells as targets. NK cells derived from CD34^+^ cells of different donors were co-incubated with the target cells for 2.5 h at an E/T ratio of 10:1. While NK cell products from two donors displayed profound cell killing activity, reaching up to 80% of target cell lysis, NK cells derived from CD34^+^ cells of two other donors tested showed no killing of the target cells under similar conditions (Figure 3B; also see Figure 7B, left panel).

### 3.3. Generation of IL-21-Expressing K562 Feeder Cells

**Figure 4:**
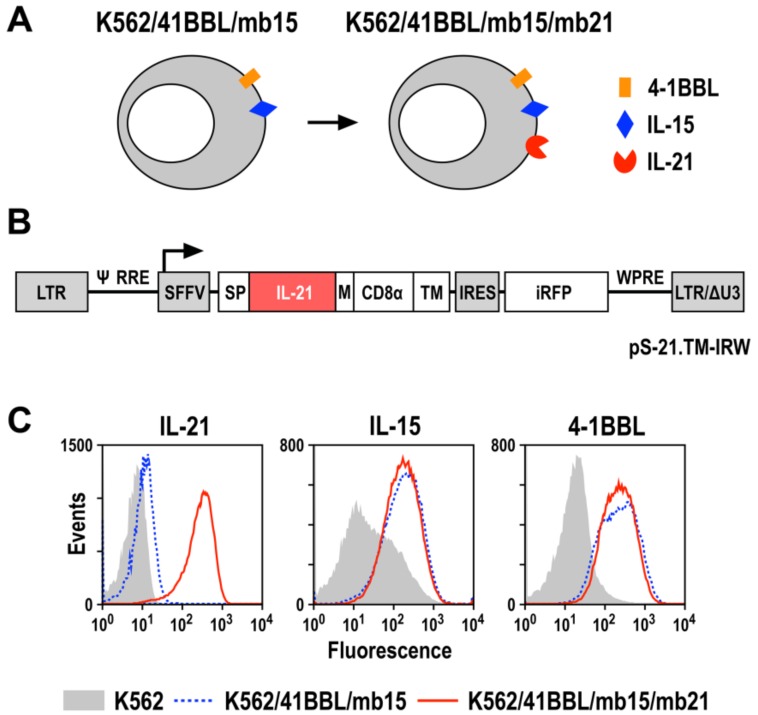
Genetically engineered K562 feeder cells for expansion of NK cells. (**A**) Schematic representation of K562/41BBL/mb15 and K562/41BBL/mb15/mb21 feeder cells that express membrane-bound pro-NK factors 4-1BB ligand (4-1BBL), IL-15 (mb15), and IL-21 (mb21). (**B**) K562/41BBL/mb15/mb21 cells were generated by transduction of previously described K562/41BBL/mb15 cells [12] with lentiviral vector pS-21.TM-IRW, which encodes under the control of the spleen focus-forming virus promoter (SFFV), a fusion of IL-21 linked via a flexible CD8α hinge region to the transmembrane and truncated intracellular domain of CD28 (TM), followed by an internal ribosome entry site (IRES) and cDNA-encoding near-infrared fluorescent protein (iRFP) as a marker. SP, immunoglobulin signal peptide; M, Myc tag; LTR, HIV-1 5’ long terminal repeat; Ψ, HIV-1 packaging signal; RRE, HIV-1 Rev response element; WPRE, woodchuck hepatitis virus post-transcriptional regulatory element; LTR/ΔU3, HIV-1 3’ LTR in self-inactivating (SIN) configuration. (**C**) After transduction, enhanced green fluorescent protein (EGFP)- and iRFP-expressing K562/41BBL/mb15/mb21 cells (red solid line) were enriched by flow cytometric cell sorting and analyzed for surface expression of IL-21, IL-15, and 4-1BBL using specific fluorochrome-conjugated antibodies. Parental K562 cells (gray shaded area) and K562/41BBL/mb15 cells (blue dotted line) served as controls.

Previously, co-culture of PBMCs with gene-modified K562 cells that expressed membrane-bound human IL-15 (mb15) and the ligand for the costimulatory receptor 4-1BB (CD137), 4-1BBL (here termed K562/41BBL/mb15 cells), was demonstrated to induce rapid NK cell expansion without or with very low T-cell proliferation [12,40]. Subsequently, a K562 variant that carried membrane-bound IL-21 (mb21) instead of mb15 was shown to be even superior in expanding NK cells [14]. Based on these studies, here we developed a K562 feeder cell line based on the K562/41BBL/mb15 cells described by Imai et al. [12], which also expresses mb21 (Figure 4A). Thereby, IL-15 and IL-21 may act synergistically to enhance NK cell expansion ex vivo. To express IL-21 on the cell surface, we constructed a lentiviral vector (pS-21.TM-IRW) encoding human IL-21 fused to the CD8α hinge region and the transmembrane domain of CD28 (21.TM) under the control of the spleen focus-forming virus promoter (Figure 4B). The vector also encodes near-infrared fluorescent protein (iRFP) as a marker linked to the 21.TM sequence via an internal ribosome entry site.

After transduction of the EGFP-positive K562/41BBL/mb15 cells with lentiviral particles, EGFP/iRFP co-expressing K562/41BBL/mb15/mb21 cells were enriched by two rounds of flow cytometric cell sorting (Supplementary Figure S2A). As a control, K562/mb21 cells were generated by lentiviral transduction of parental K562 cells and enrichment of iRFP-expressing cells. The IL-21 fusion protein with an apparent molecular mass of 30 kDa was readily detected by immunoblot analysis of K562/41BBL/mb15/mb21 and K562/mb21 cell lysates, with a CD8α-specific antibody recognizing the hinge region of the molecule (Supplementary Figure S2B), verifying the expression and integrity of the chimeric protein. In addition, surface expression of IL-21 was confirmed in the sorted K562/41BBL/mb15/mb21 cells by flow cytometry using an IL-21-specific antibody (Figure 4C). Thereby, lentiviral transduction and IL-21 expression did not alter surface expression of IL-15 or 4-1BBL, as confirmed by comparison of K562/41BBL/mb15/mb21 to the original K562/41BBL/mb15 cells (Figure 4C).

Binding of IL-21 to its cognate receptor (IL-21R) on target cells induces the phosphorylation of STAT molecules, with STAT3 shown to be the dominant signal transducer following receptor engagement [41]. Hence, to investigate functionality of the membrane-anchored IL-21 protein, we co-incubated IL-21R-expressing Raji cells with K562/41BBL/mb15/mb21 cells and analyzed STAT3 activation by immunoblotting (Supplementary Figure S3). Thereby, both isoforms of phosphorylated STAT3 (pSTAT3α and β) were readily detected in Raji cell lysates after exposure to K562/41BBL/mb15/mb21 cells, but not if Raji cells were co-cultured with K562/41BBL/mb15 or parental K562 cells.

### 3.4. Expansion of NK Cells from Peripheral Blood with K562/41BBL/mb15/mb21 Feeder Cells

**Figure 5:**
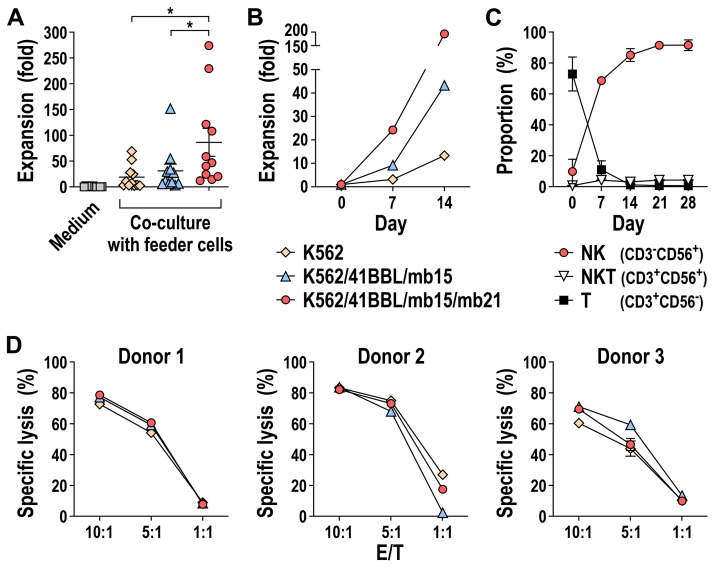
Selective expansion of CD56^+^ NK cells from peripheral blood with K562/41BBL/mb15/mb21 feeder cells. Peripheral blood mononuclear cells (PBMCs) from healthy donors were mixed with lethally irradiated K562/41BBL/mb15/mb21 or K562/41BBL/mb15 cells at a ratio of 1.5:1 (PBMCs/feeder cells) and cultured in medium containing 50 IU/mL of IL-2. For comparison, NK cells from peripheral blood were expanded using either irradiated parental K562 cells or only IL-2-containing culture medium. (**A**) Expansion of CD56^+^CD3^-^ NK cells after seven days of co-culture with different feeder cells relative to the number of input cells (day 0) was analyzed by flow cytometry using specific fluorochrome-conjugated antibodies. Note: *n* = 11 individual donors. Individual data points and mean values ± SEM are shown; * *p* < 0.05. (**B**) On day 7, expanded NK cells were restimulated with the respective feeder cells, and expansion of NK cells on day 14 was determined as described in (A). Data for a representative donor are shown. (**C**) Immune cell composition (NK, NKT, and T cells) in K562/41BBL/mb15/mb21 feeder cell-stimulated cell pools was determined over time by flow cytometry using CD56- and CD3-specific antibodies. Mean values ± SEM are shown; *n* = 2 individual donors. (**D**) Cytotoxicity of CD56^+^ NK cells expanded from PBMCs of three individual donors using the feeder cells indicated in (A) and (B) against K562 cells was determined in flow-cytometry-based cytotoxicity assays at different effector to target (E/T) ratios, as indicated. E/T ratios were calculated based on the proportion of CD56^+^ NK cells. Mean values ± SEM of three technical replicates for each donor are shown.

First, we investigated the ability of K562/41BBL/mb15/mb21 cells to facilitate expansion of peripheral blood NK cells in comparison to K562/41BBL/mb15. Freshly isolated PBMCs from healthy donors were cultured with lethally irradiated K562/41BBL/mb15/mb21 or K562/41BBL/mb15 cells at a ratio of 1.5:1 (PBMCs/feeder cells) in medium containing 50 IU/mL IL-2. PBMCs co-cultured with irradiated parental K562 cells or cultured in IL-2-containing medium alone were included as controls. Expansion over time was normalized to the proportion of NK cells (CD56^+^CD3^-^) in each starting PBMC product at day 0. Co-culture with K562/41BBL/mb15/mb21 cells resulted in a significantly greater expansion of NK cells after 7 days (mean 86-fold; 11 donors) when compared to K562/41BBL/mb15 cells (mean 31-fold) and parental K562 cells (mean 19-fold), while culture of PBMCs without feeder cells in medium containing only IL-2 induced no significant NK cell expansion (mean 0.75-fold) (Figure 5A). Culture conditions were maintained for up to 4 weeks, with restimulation of expanding NK cells with the respective feeder cells every 7 days. Thereby, K562/41BBL/mb15/mb21-activated NK cells exhibited the most pronounced growth and proliferation (shown for a representative donor in Figure 5B), with a mean expansion of 372-fold observed after 14 days (*n* = 6), and 842-fold after 21 days (*n* = 4) (data not shown). Furthermore, in K562/41BBL/mb15/mb21-stimulated cell pools, the increase in the NK cell population over time was accompanied by a concomitant decline in the T-cell proportion, with a very low expansion of NKT cells (Figure 5C). The mean percentage of CD56^+^CD3^-^ NK cells in the cultures was 68.6% at day 7, 85.2% at day 14, 91.5% at day 21, and 91.6% at day 28, while that of CD56^-^CD3^+^ T cells was 11%, 1.2%, 0.8%, and 0.6%; and that of CD56^+^CD3^+^ NKT cells 4.2%, 3.1%, 4.3%, and 4.3% on the same days, respectively (*n* = 2).

We also examined expression of various NK-cell-associated cell surface markers in the different feeder cell-expanded NK cell pools (Supplementary Figure S4A). Thereby, the proportion of NKG2D-expressing cells was about 50% lower among K562/41BBL/mb15/mb21-activated CD56^+^CD3^-^ NK cells than among NK cells cultured with K562/41BBL/mb15 cells, while the proportion of CD16-positive cells was overall very low but comparable between the different culture conditions. Also the proportion of NK cells expressing the natural cytotoxicity receptors NKp30 and NKp44 was considerably lower among K562/41BBL/mb15/mb21-activated NK cells, while irrespective of the culture conditions, more than 80% of the cells expressed NKp46. Likewise, we observed no difference in the proportion of cells expressing the inhibitory receptor complex NKG2A/CD94, whereas fewer cells expressed KIR2D receptors upon co-culture with K562/41BBL/mb15/mb21 cells. However, despite the lower expression of several activating receptors by K562/41BBL/mb15/mb21-expanded NK cells, their cytotoxicity against K562 target cells at different E/T ratios was comparable to that of K562/41BBL/mb15-expanded peripheral blood NK cells (Figure 5D).

### 3.5. Effect of K562/41BBL/mb15/mb21 Feeder Cells on Ex Vivo NK Cell Generation from Mobilized CD34^+^ Progenitors

**Figure 6:**
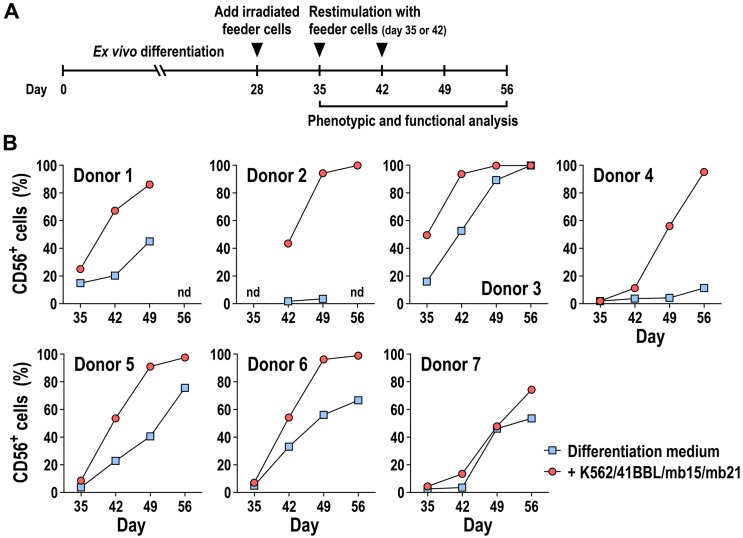
Ex vivo NK cell generation from CD34^+^ progenitors in the presence of gene-modified K562 cells. (**A**) Schematic representation of the procedure used for ex vivo generation of NK cells from mobilized PB-CD34^+^ HSCs with gene-modified K562 feeder cells. PB-CD34^+^ cells were first cultured for four weeks in cytokine-containing medium following the protocol described in Figure 1A. On day 28, 2 × 10^6^ cells from the differentiating cell pool were mixed with 1 × 10^6^ lethally irradiated K562/41BBL/mb15/mb21 feeder cells. Starting after one week of co-culture (day 35), the development of CD56^+^ NK cells was analyzed once weekly by flow cytometry. Ex-vivo-generated CD56^+^ NK cells were restimulated with K562/41BBL/mb15/mb21 cells, as described in Materials and Methods. (**B**) Generation of CD56^+^ NK cells from PB-CD34^+^ cells following stimulation with K562/41BBL/mb15/mb21 feeder cells (*n* = 7 individual donors). For comparison, CD34^+^ cells cultured in regular differentiation medium without feeder cells were included (data for these controls are the same as shown in Figure 3A). Note: nd = not determined.

Next, we analyzed the influence of K562/41BBL/mb15/mb21 feeder cells on the ex vivo generation of NK cells from mobilized CD34^+^ progenitors. To allow direct comparison, we used the same donor cells employed before for only cytokine-based expansion and differentiation (shown in Figure 3). Mobilized PB-CD34^+^ cells were first cultured following the ex vivo expansion–differentiation protocol with cytokine-containing media. Subsequently, at day 28 of the culture period, lethally irradiated K562/41BBL/mb15/mb21 cells were added to the differentiating cultures, and development of CD56^+^ NK cells over time was tracked by flow cytometry (Figure 6A). In contrast to differentiation with cytokines alone, additional stimulation with K562/41BBL/mb15/mb21 cells resulted in superior and faster expansion of CD56^+^ NK cells (Figure 6B). For 5 out of 7 donors tested, the difference in NK cell yield was already apparent after co-culture with K562/41BBL/mb15/mb21 cells for two weeks. Furthermore, feeder-cell stimulated cells continued to grow rapidly and yielded a very pure CD56^+^ NK cell population that routinely reached >99% of CD56^+^ cells in the final culture (day 56). Importantly, addition of K562/41BBL/mb15/mb21 cells also allowed efficient NK cell development from donor CD34^+^ cells that displayed poor differentiation capacity in the cytokine-based culture scheme only (Figure 6B, donors 2 and 4). 

### 3.6. Phenotype and Cytotoxic Activity of HSC-Derived NK Cells Stimulated with K562/41BBL/mb15/mb21 Feeder Cells

**Figure 7:**
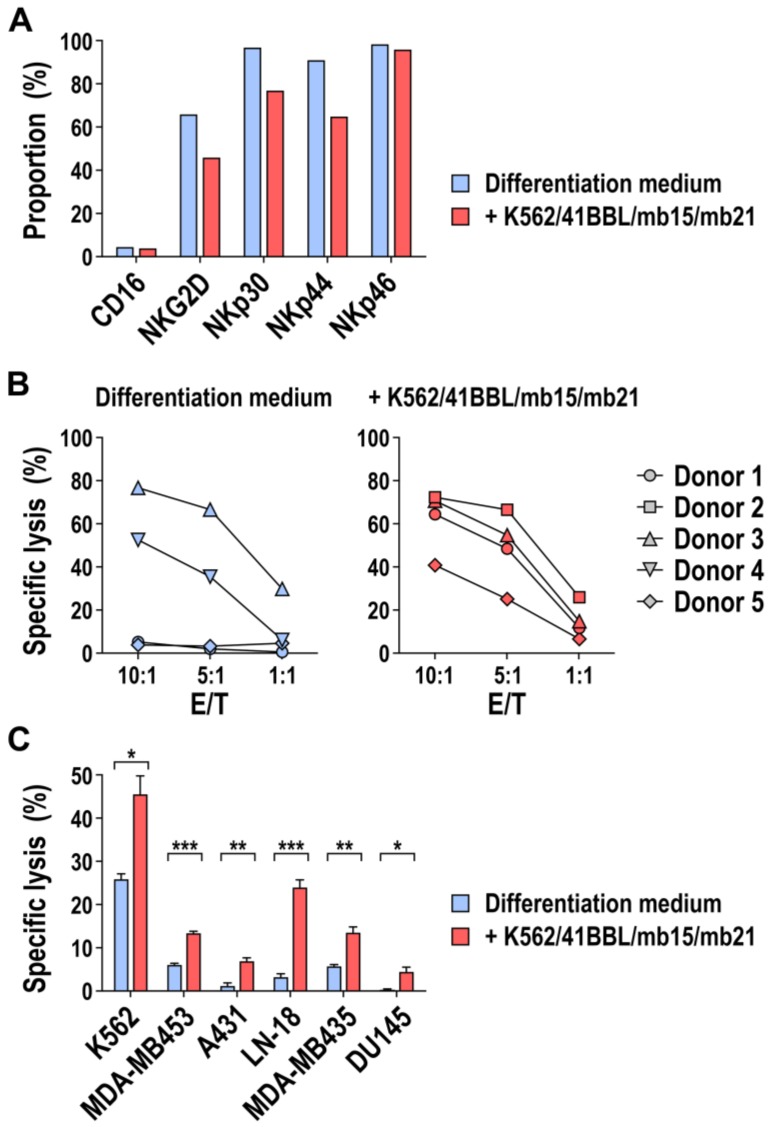
Phenotypic and functional characterization of ex-vivo-generated CD56^+^ NK cells co-cultured with K562/41BBL/mb15/mb21 feeder cells. (**A**) Surface expression of NK-cell-associated activating receptors CD16, NKG2D, and NCRs (NKp30, NKp44, NKp46) by ex-vivo-generated CD56^+^ cells after co-culture with K562/41BBL/mb15/mb21 feeder cells (red bars) or cultured in regular cytokine-containing medium (blue bars) was analyzed by flow cytometry. Results from a representative experiment are shown. (**B**) Cytotoxicity of CD56^+^ NK cells generated from PB-CD34^+^ cells using K562/41BBL/mb15/mb21 feeder cells towards K562 leukemia cells was determined in flow-cytometry-based cell killing assays at different effector to target (E/T) ratios (right panel). NK cells generated only using cytokine-containing medium were included as controls (left panel). Cells from donors 1, 3, and 5 were tested in parallel using both conditions, while cells from donors 2 and 4 were only expanded using K562/41BBL/mb15/mb21 feeder cells or cytokine-containing medium, respectively. Mean values ± SEM of three technical replicates for each donor are shown. Donors are the same as for the experiments shown in Figure 3. (**C**) MDA-MB453 breast carcinoma, A431 squamous cell carcinoma, LN-18 glioblastoma, MDA-MB435 melanoma, and DU145 prostate cancer cells were co-incubated with CD56^+^ NK cells either generated with K562/41BBL/mb15/mb21 feeder cells (red bars) or only with cytokine-containing medium (blue bars) for 2.5 h at an E/T ratio of 5:1, and NK-cell-mediated cytotoxicity was determined by flow cytometry. K562 leukemia cells were included for comparison. Mean values ± SEM of three technical replicates performed with the cells of a representative donor are shown; *** *p* < 0.001; ** *p* < 0.01; * *p* < 0.05.

To assess whether the striking difference in the extent of CD56^+^ NK cells generated from mobilized HSCs in the absence or presence of K562/41BBL/mb15/mb21 feeder cells also resulted in phenotypic differences between the cells, we compared expression of NK-cell-associated cell surface markers by flow cytometry. Thereby, we found that NK cells exposed to K562/41BBL/mb15/mb21 cells displayed markedly lower expression of NKG2D, NKp30, and NKp44 than NK cells differentiated from PB-CD34^+^ cells and only expanded in cytokine-containing medium, while expression of CD16 and NKp46 was comparable between both cell populations (Figure 7A). These findings were consistent with the results obtained with peripheral blood NK cells expanded with K562/41BBL/mb15/mb21 feeder cells (see Supplementary Figure S4A).

To examine potential functional differences between NK cells differentiated from HSCs and exposed to K562/41BBL/mb15/mb21 feeder cells and ex-vivo-generated NK cells only kept in cytokine-containing medium, we first performed cytotoxicity assays using K562 cells as targets. CD56^+^ NK cells differentiated from mobilized HSCs of four independent donors were co-incubated with target cells for 2.5 h at different E/T ratios. For a consistent comparison between ex-vivo-generated NK cells exposed to K562/41BBL/mb15/mb21 feeder cells and NK cells derived only by culture in cytokine-containing medium, the E/T ratio was calculated based on the proportion of CD56^+^ NK cells and not the total number of cells in the cultures. As shown in Figure 7B, target cell lysis by NK cells differentiated and expanded using only cytokines was extremely variable, with NK cells from two donors efficiently killing K562 target cells, while cells from the other two donors tested showed no cytotoxicity, even at the relatively high E/T ratio of 10:1 (range 3.8–76.5% of cell killing). In contrast, ex vivo differentiated NK cells that were exposed to K562/41BBL/mb15/mb21 feeder cells during culture exhibited consistently high cytotoxicity against K562 target cells (range 40.6–72.1% of cell killing at an E/T ratio of 10:1), even in the case of donors whose NK cells were inactive if generated only with cytokine-containing medium (Figure 7B, donors 1 and 5).

While MHC class I negative K562 cells are typically very sensitive to NK cells, cancer cells of various solid tumor origins can display partial or complete resistance in short-term cytotoxicity assays. Hence, we also tested the cell killing activity of NK cells generated from mobilized HSCs and expanded with K562/41BBL/mb15/mb21 feeder cells against MDA-MB453 breast carcinoma, A431 squamous cell carcinoma, LN-18 glioblastoma, MDA-MB435 melanoma, and DU145 prostate carcinoma cells as targets. Thereby, the different tumor cells tested all proved sensitive to ex-vivo-generated and K562/41BBL/mb15/mb21-expanded NK cells upon 2.5 h of co-culture at an E/T ratio of 5:1, albeit to a lesser extent than K562 cells, which were again included for comparison (Figure 7C). Importantly, in all cases, cytotoxicity of K562/41BBL/mb15/mb21-expanded NK cells was markedly enhanced in comparison to the cell killing activity of HSC-derived NK cells from the same donor which were differentiated and expanded only with cytokines. Furthermore, K562/41BBL/mb15/mb21-expanded NK cells produced markedly enhanced levels of interferon (IFN)-γ, tumor necrosis factor (TNF)-α, and granulocyte-macrophage colony-stimulating factor (GM-CSF) after stimulation with K562 target cells than NK cells generated from mobilized HSCs only with cytokine-containing differentiation medium (Supplementary Figure S5).

## 4. Discussion

The potential of donor-derived natural killer cells for adoptive cancer immunotherapy is increasingly being recognized. Such approaches strongly rely on the availability of sufficient numbers of functional effector cells to achieve a clinical response. Hence, significant efforts have been made in recent years to establish ex vivo culture protocols that support robust and large-scale expansion of activated NK cells from the peripheral blood of healthy donors or different types of hematopoietic stem and progenitor cells [7,8,9,10,11]. To overcome limitations of current strategies for ex vivo differentiation of NK cells from G-CSF-mobilized peripheral blood CD34^+^ cells, here we developed an optimized culture protocol that reproducibly allows the generation of functionally mature NK cells. We implemented an enriched cocktail of cytokines and growth factors to facilitate PB-CD34^+^ cell expansion and subsequent differentiation into NK cells. To further aid NK cell development, we included K562 feeder cells in the protocol, that in addition to 4-1BBL and membrane-bound IL-15 were genetically engineered to express a membrane-anchored derivative of IL-21, which counteracted remaining donor-to-donor variability with respect to the yield and antitumor activity of the generated NK cells.

Mobilized PB-CD34^+^ cells are a readily accessible source of stem cells and are routinely employed in allogeneic hematopoietic stem cell transplantation. Post infusion and engraftment, PB-CD34^+^ cells can reconstitute a complete immune system in the recipient, including the development of NK cells. However, the use of PB-CD34^+^ cells for ex vivo generation of NK cells remains challenging. Previous studies have tested different culture conditions, including diverse combinations of cytokines, growth factors, and stromal cells as feeder layers to commit stem cells to the NK lineage [22,29,30,31,32,42]. Overall, NK cell development in these studies appeared inefficient and highly variable, often yielding effector cells of low purity or lacking adequate cytolytic activity. This may in part be attributed to the G-CSF used as a mobilizing agent for the collection of PB-CD34^+^ cells, which preferentially generates myeloid-committed precursors. Moretta and colleagues showed that continued or even transient exposure of CD34^+^ cells to G-CSF substantially inhibited their ex vivo differentiation into innate lymphoid cells (ILCs), which also include NK cells, irrespective of whether peripheral blood or cord blood were used as the stem cell source [32]. Hence, to limit development of myeloid cells during the differentiation process and facilitate preferential NK cell generation, we incorporated hydrocortisone in the cytokine cocktail used for differentiation. Physiological concentrations of HDC have been shown to advance NK cell development from CD34^+^ progenitors [38], and along with cytokines and stromal cells, HDC can enhance NK cell differentiation by recruiting myeloid precursors (CMP) and progenitors (GMP) to the NK lineage [39]. We also included insulin-like growth factor-1 in the differentiation medium, which was previously shown to promote development of NK cells from cord blood derived CD34^+^ cells by upregulating NFIL3 (E4BP4) and ID2, two critical transcription factors involved in NK cell development [37,43,44]. In combination with IL-15, IGF-1 can also improve NK cell cytotoxicity through activation of the STAT3 pathway and subsequent upregulation of the effector molecule perforin [37].

Human plasma is a rich source of proteins and factors that play an important role in the maintenance, growth, and proliferation of cells. To assess the influence of plasma on NK cell development, we initially performed parallel differentiation experiments with PB-CD34^+^ cells either cultured in cytokine-supplemented serum-free or serum-rich media (data not shown). Thereby, substantial CD56^+^ populations were observed only in the presence of human plasma, indicating that it may be indispensable for efficient NK cell development from mobilized HSCs. With the exception of mobilized HSCs from two donors (poor responders), all other donor PB-CD34^+^ cells tested in this study efficiently differentiated and gave rise to NK cells upon culture in cytokine-enriched and plasma-containing medium. This indicates that the established ex vivo differentiation protocol is overall robust and reliable, and has the potential to produce NK cells in a consistent manner. The resulting NK cells displayed a mature and activated phenotype, indicated by high and stable expression of CD56; the activating receptors NKG2D, NKp44, and NKp46; and the inhibitory receptors NKG2A and CD94 [45]. We also found expression of NKp30, but there was considerable variation in the proportion of NKp30-positive cells between the different donors. Likewise, expression of CD16 and KIR molecules was either absent or very low, reflecting observations made in previous studies [20,22,46].

In the two cases where NK cell yields were low after differentiation in cytokine-enriched medium, initial G-CSF-induced mobilization of mostly myeloid-committed precursors may be responsible. This is supported by the observation that the majority of cells in cultures from the poor responders were CD33-negative but expressed the myeloid markers CD13, CD14, and CD15 (data not shown), indicating preferential differentiation of the PB-CD34^+^ cells into the myeloid lineage. CD34^+^ cells from these donors may have had a high proportion of myeloid-committed progenitors already in later stages of development, which could no longer be dedifferentiated or recruited into the NK cell pathway, even with hydrocortisone-supplemented medium. Despite having successfully differentiated into NK cells, cells from two other donors tested did not display substantial cytotoxicity towards K562 target cells. The reason for the lack of cell killing activity by these NK cells is unclear at present. While prolonged stimulation with IL-15 has been reported to induce exhaustion of NK cells [47], this may not be a general effect, as suggested by studies with established NK cell lines and donor-derived primary NK cells, which retained activity despite continuous autocrine stimulation through IL-15 ectopically expressed by the NK cells after transduction with viral vectors [48,49].

Co-culture with irradiated feeder cells has been described by different groups to enhance ex vivo NK cell expansion and activation. Imai and colleagues first demonstrated specific and vigorous expansion of NK cells (median > 1000-fold at 3 weeks) upon culture of PBMCs with K562 cells that were genetically engineered to express membrane-bound IL-15 (mb15) and 4-1BBL (K562-mb15-41BBL cells; in our study designated as K562/41BBL/mb15) [12]. Replacing mb15 with IL-21 (mb21) further enhanced this proliferation-inducing capacity, with a median 31,747-fold expansion of NK cells reported at 3 weeks of culture [14]. Moreover, NK cells expanded with mb21-expressing K562 cells displayed enhanced cytokine secretion and longer telomeres, and were less prone to exhaustion or senescence. Based on the latter report and earlier studies showing a synergistic effect of recombinant IL-15 and IL-21 on proliferation, cytokine secretion, and cytotoxicity of NK cells [14,50,51], here we modified the K562 feeder cells reported by Imai et al. to express a membrane-anchored form of IL-21 (mb21) in addition to mb15 and 4-1BBL. In line with the previous studies, the resulting K562/41BBL/mb15/mb21 cells induced significantly greater expansion of NK cells from donor PBMCs when compared to K562/41BBL/mb15 cells, with a mean 842-fold NK cell expansion observed after three weeks of culture. We did not find proliferation of T and B cells, and only low-level expansion of NKT cells, indicating selectiveness of the feeder cells’ stimulatory effects towards NK cells. We also generated and tested a K562-based feeder cell line that only expressed mb21 (K562/mb21 cells; see Supplementary Figure S2B). However, in contrast to K562/41BBL/mb15/mb21, these cells were ineffective in stimulating NK cell expansion (data not shown).

In comparison to K562/41BBL/mb15-expanded NK cells, NK cells stimulated by K562/41BBL/mb15/mb21 cells demonstrated decreased surface expression of KIR molecules and the activating receptors NKG2D, NKp30, and NKp44, while expression of the activating receptor NKp46 and the inhibitory receptor complex NKG2A/CD94 was similar. These differences in surface receptor expression did not impair cytotoxic activity of the K562/41BBL/mb15/mb21-expanded NK cells, indicating that their higher proliferation rate did not compromise functionality. Interestingly, K562/41BBL/mb15/mb21-expanded NK cells displayed increased expression of granzyme B (see Supplementary Figure S4B), which corresponds well with a previous report demonstrating upregulation of perforin and granzymes upon treatment of NK cells with recombinant IL-21 [52].

Encouraged by the promising results obtained with peripheral blood NK cells, we explored the suitability of K562/41BBL/mb15/mb21 feeder cells to further improve ex vivo differentiation of NK cells from mobilized HSCs. To allow a reliable comparison, side-by-side experiments with the same donor PB-CD34^+^ cells were performed either in the absence (regular cytokine medium) or presence of feeder cells. Indeed, addition of K562/41BBL/mb15/mb21 cells to differentiating cultures markedly improved ex vivo generation of NK cells from HSCs. Thereby, highly pure (>99%) NK cell products were obtained. Consistent with the findings with peripheral blood NK cells, PB-CD34^+^-derived NK cells differentiated and expanded in the presence of K562/41BBL/mb15/mb21 feeder cells displayed lower surface expression of the activating receptors NKG2D, NKp30, and NKp44 than NK cells exposed only to cytokine-containing medium, while expression of CD16 and NKp46 were comparable. Importantly, irradiated K562/41BBL/mb15/mb21 cells also promoted NK cell development from PB-CD34^+^ cells that displayed poor differentiation capacity in regular cytokine-based culture, demonstrating that this approach can overcome the donor-to-donor variability often observed with G-CSF-mobilized HSCs. Expression of membrane-anchored IL-21 by the feeder cells strongly contributed to this effect, since K562/41BBL/mb15 cells were less effective than K562/41BBL/mb15/mb21 in generating NK cells from the same donor PB-CD34^+^ cells (data not shown).

In cell killing experiments, NK cells differentiated from HSCs of all tested donors that were expanded in the presence of K562/41BBL/mb15/mb21 feeder cells exhibited marked activity and displayed equal or greater cytotoxicity against K562 target cells than NK cells only differentiated and expanded in cytokine-containing medium. Importantly, as seen for the extent of differentiation of NK cells from poor responders, PB-CD34^+^ cells of two donors that gave rise to NK cells without measurable cytotoxicity if generated only with cytokines yielded NK cells that efficiently killed K562 cells when stimulated with K562/41BBL/mb15/mb21 cells during differentiation and expansion. This differential activity of NK cells derived from the same donor HSCs may be attributed to the previously described ability of IL-21 to reverse exhaustion and restore antitumor activity of NK cells after long-term culture [14,53]. Furthermore, in addition to killing of K562 cells, we observed enhanced lysis of target cells of different solid tumor origins by NK cells generated with the help of K562/41BBL/mb15/mb21 feeder cells.

Taken together, our data demonstrate that mobilized hematopoietic stem and progenitor cells expanded and differentiated according to this two-step protocol are a promising source for the generation of allogeneic NK cells. Thereby, exposure to genetically engineered K562 feeder cells expressing membrane-anchored IL-15 and IL-21 counteracts donor-to-donor variability observed with cytokine-based differentiation only, and drives rapid and robust expansion of highly pure NK cells with an activated phenotype. Such cells may also serve as a basis for genetic modification to further augment their antitumor activity [54,55,56], which will be explored in subsequent studies.

## Supplementary Materials

The following are available online at https://www.mdpi.com/2073-4409/9/4/811/s1: Figure S1: Ex vivo generation of NK cells from bone marrow-derived hematopoietic progenitors. Figure S2: Expression of IL-21 by genetically modified K562/41BBL/mb15/mb21 feeder cells. Figure S3: Activation of STAT3 upon exposure of Raji cells to K562/41BBL/mb15/mb21 feeder cells. Figure S4: Phenotypic characterization of peripheral blood-derived NK cells. Figure S5: Secretion of pro-inflammatory cytokines by ex-vivo-differentiated NK cells.
