# Supplementary material for: Directed Differentiation of Mobilized Hematopoietic Stem and Progenitor Cells into Functional NK Cells with Enhanced Antitumor Activity
Source: Cells. 2020 Mar 27;9(4):811. doi: 10.3390/cells9040811 (PMC7226771; doi:10.3390/cells9040811)
Supplement: Supplementary file 1 [file cells-09-00811-s001.pdf]

# Directed Differentiation of Mobilized Hematopoietic Stem and Progenitor Cells into Functional NK cells with Enhanced Antitumor Activity

**Pranav Oberoi, Kathrina Kamenjarin, Jose Francisco Villena Ossa, Barbara Uherek, Halvard Bönig and Winfried S. Wels**

## Supplementary data

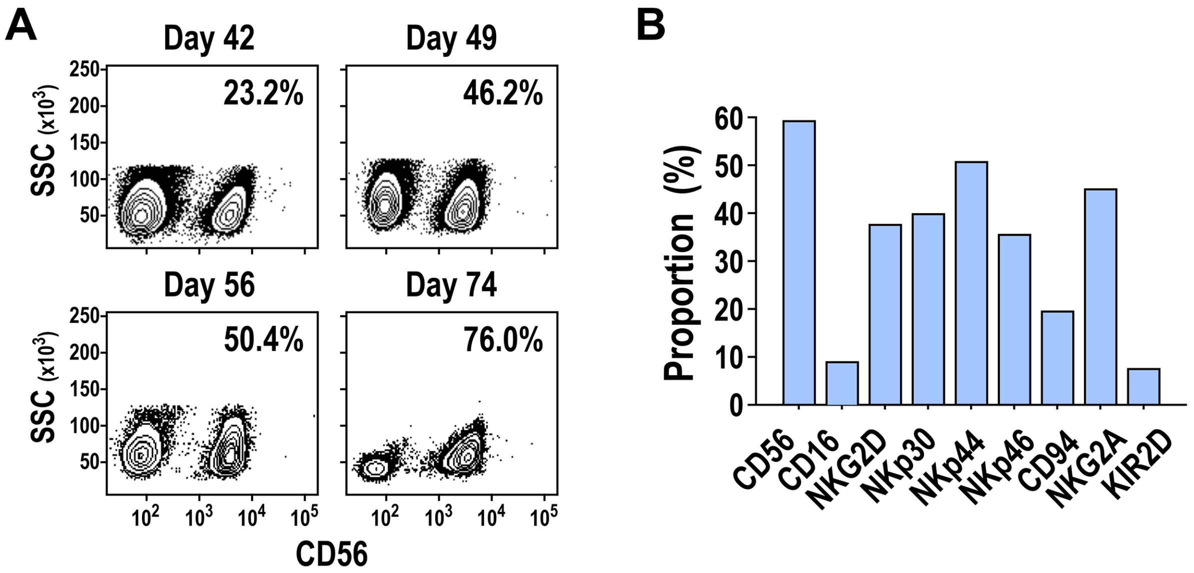


**Supplementary Figure S1.** *Ex vivo* generation of NK cells from bone marrow-derived hematopoietic progenitors. (**A**) $CD34^+$ cells were isolated from bone marrow (BM) aspirate of a healthy donor employing the same selection protocol as described in Materials and Methods for mobilized PB-$CD34^+$ cells. Enriched BM-$CD34^+$ cells were subsequently cultured following the scheme depicted in Figure 1A. Development of $CD56^+$ NK cells was analyzed at the indicated time points of the culture period by flow cytometry. (**B**) Surface expression of NK-cell-associated receptors by cells generated *ex vivo* from BM-$CD34^+$ cells was analyzed by flow cytometry (cells from day 67 of the culture).

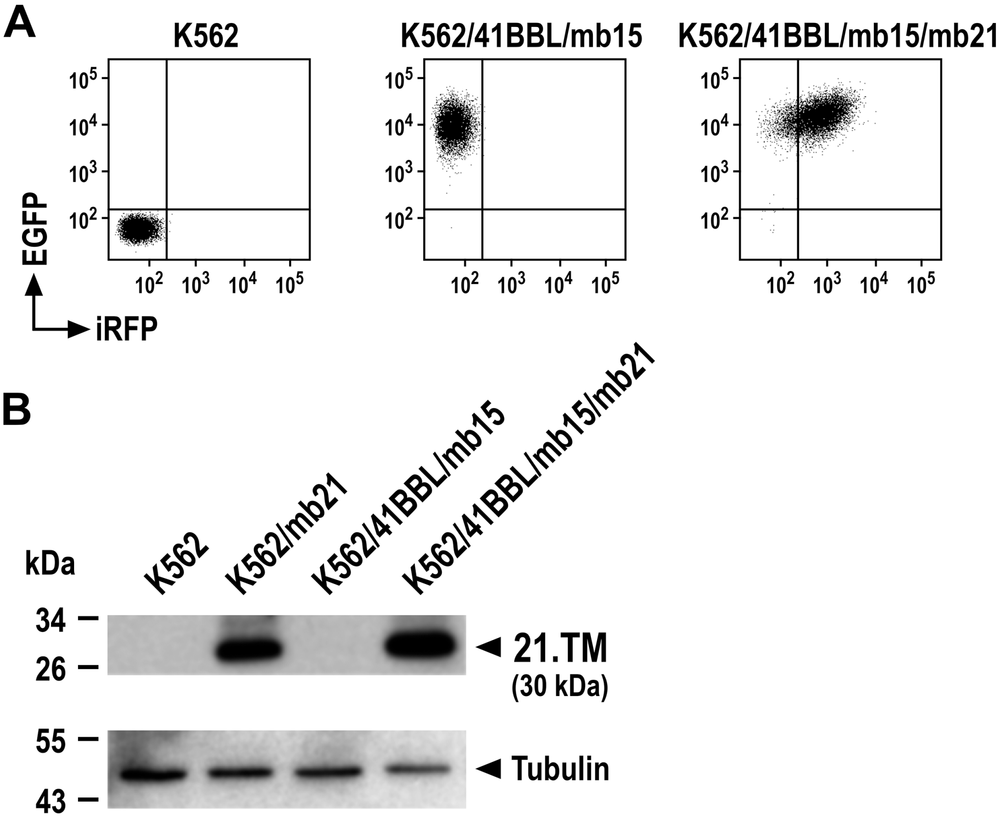


**Supplementary Figure S2.** Expression of IL-21 by genetically modified K562/41BBL/mb15/mb21 feeder cells. (**A**) After transduction of EGFP-positive K562/41BBL/mb15 cells (middle panel) with pS-21.TM-IRW lentiviral vector encoding membrane-anchored IL-21 and iRFP as a marker, EGFP/iRFP-double positive K562/41BBL/mb15/mb21 cells were enriched by flow cytometric cell sorting and re-analyzed for EGFP and iRFP expression (right panel). Parental K562 cells served as control (left panel). (**B**) Expression of membrane-anchored IL-21 (21.TM) by sorted K562/41BBL/mb15/mb21 cells was investigated by immunoblot analysis of cell lysates with a CD8$\alpha$-specific antibody detecting the hinge region of the molecule. Parental K562 cells, K562 cells transduced with the pS-21.TM-IRW vector and K562/41BBL/mb15 cells were included as controls. $\gamma$-Tubulin was analyzed as a loading control.

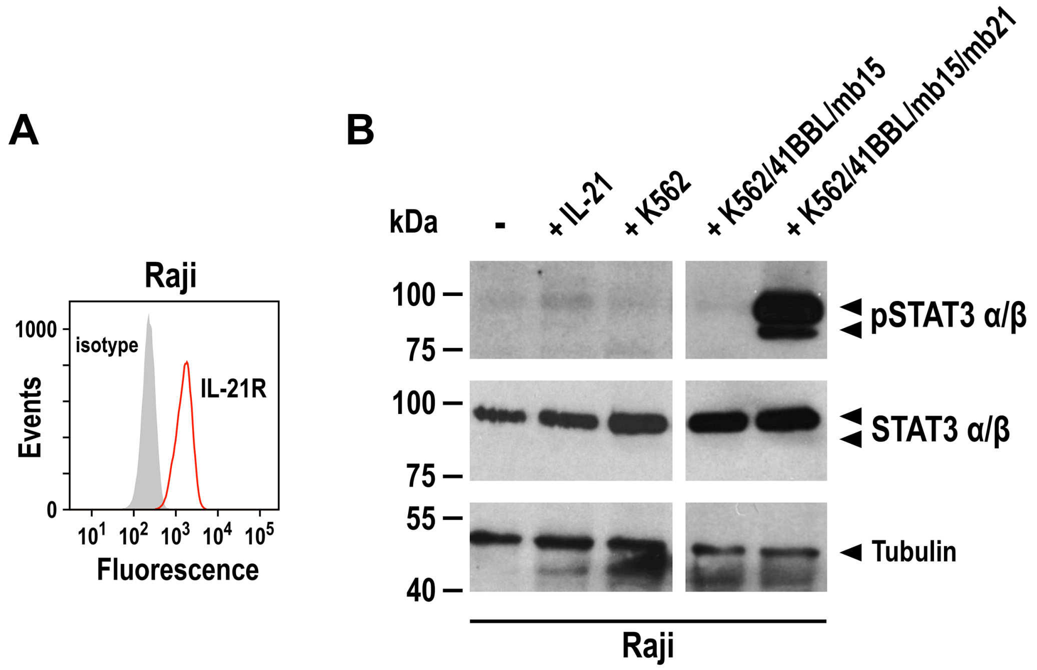


**Supplementary Figure S3.** Activation of STAT3 upon exposure of Raji cells to K562/41BBL/mb15/mb21 feeder cells. (**A**) Expression of IL-21 receptor (IL-21R) on the surface of Raji Burkitt's lymphoma cells was determined by flow cytometry with fluorochrome-conjugated IL-21R-specific antibody (red solid line). Cells incubated with an isotype-matched control antibody (gray shaded area) served as control. (**B**) To determine IL-21 activity, Raji cells were incubated with K562/41BBL/mb15/mb21, K562/41BBL/mb15 or parental K562 cells at a 1:1 ratio for 30 minutes at 37°C. Raji cells stimulated with 10 ng/mL of recombinant IL-21 or left unstimulated were included as controls. Subsequently, cell lysates were prepared and subjected to immunoblot analysis using either anti-phospho-STAT3 (pSTAT3) or anti-STAT3 primary antibody followed by HRP-coupled secondary antibody and chemiluminescent detection. γ-Tubulin was analyzed as a loading control.

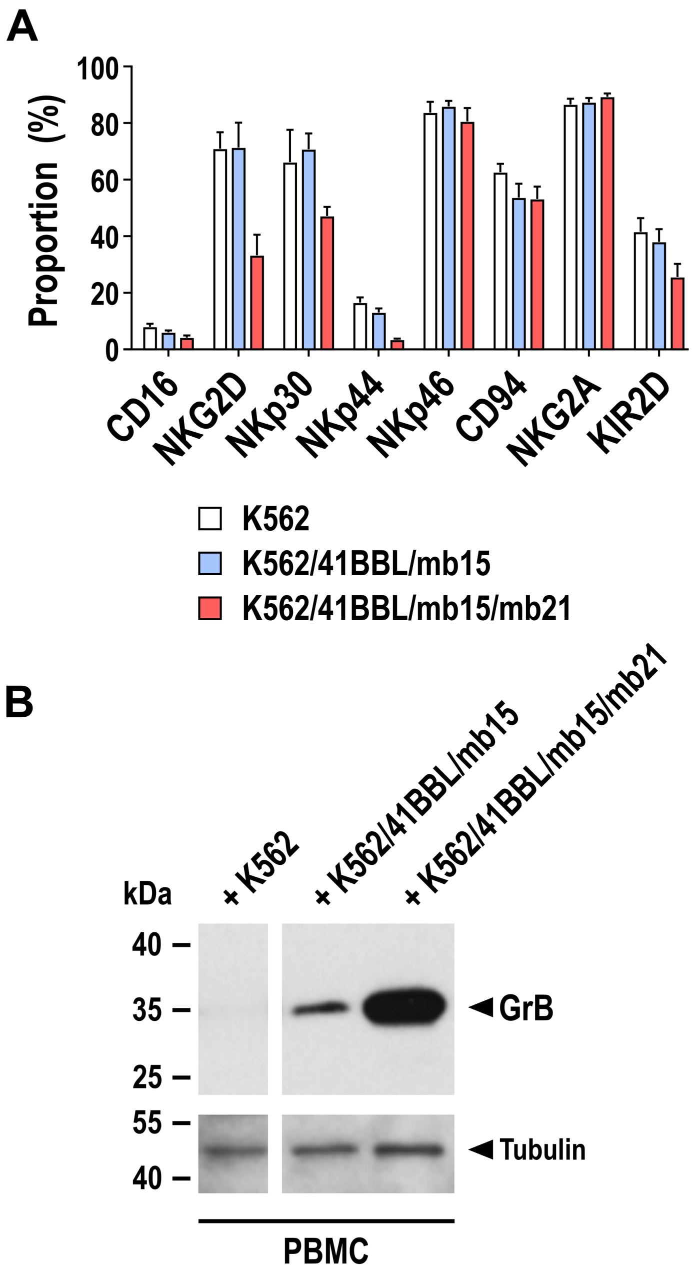


**Supplementary Figure S4.** Phenotypic characterization of peripheral blood-derived NK cells. (**A**) Surface expression of NK-cell associated receptors by $CD56^{+}CD3^{-}$ cells expanded from PBMCs of healthy donors following seven days of co-culture with the indicated K562 feeder cells was analyzed by flow cytometry. Mean values ± SEM are shown; n=9 individual donors. (**B**) Expression of granzyme B (GrB) by cells stimulated with the indicated feeder cells was investigated by immunoblot analysis of cell lysates with GrB-specific antibody. γ-Tubulin was analyzed as a loading control.

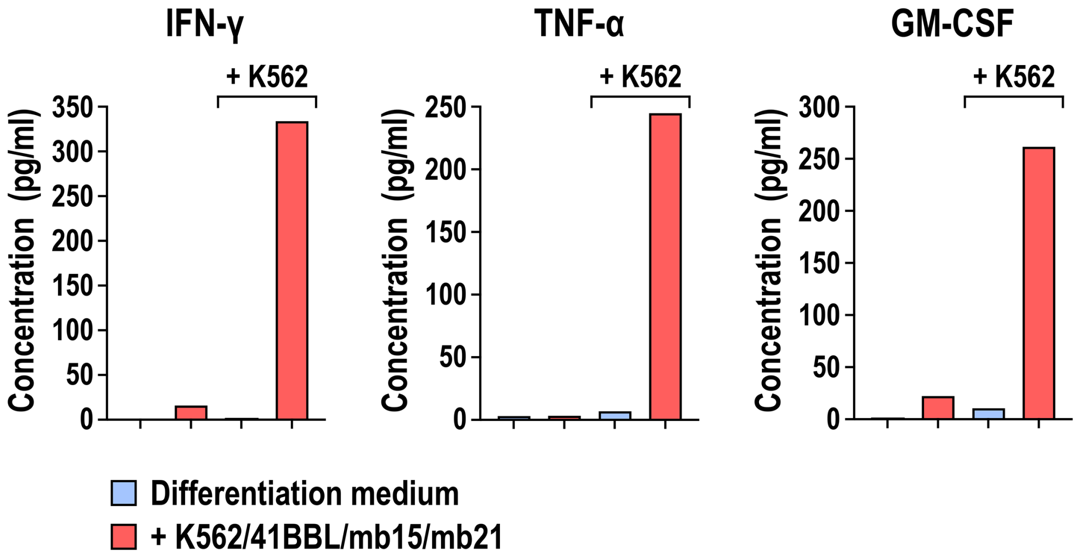


**Supplementary Figure S5.** Secretion of pro-inflammatory cytokines by *ex vivo* differentiated NK cells. CD56$^+$ NK cells (5 x $10^5$) generated *ex vivo* from mobilized PB-CD34$^+$ cells by co-culture with K562/41BBL/mb15/mb21 feeder cells (red bars) or culture in cytokine-containing medium (blue bars) were incubated for 6 hours with K562 target cells at an effector to target (E/T) ratio of 1:1 in 1 mL of regular differentiation medium. Supernatants were collected, and the levels of interferon (IFN)-$\gamma$, tumor necrosis factor (TNF)-$\alpha$, and GM-CSF were determined using a BD Cytometric Bead Array and BD FACSArray bioanalyzer (BD Biosciences) according to the manufacturer's recommendations. PB-CD34$^+$-derived NK cells kept without target cells were included as controls. Representative results obtained with cells from the same donor as in Figure 7C are shown.
